# University Clinique—Cases and Observations in Surgery

**Published:** 1854-02

**Authors:** S. D. Gross

**Affiliations:** Professor of Surgery in the University of Louisville


### Art. III.—UNVERSITY CLINIQUE—CASES AND OBSERVATIONS IN SURGERY.

BY S. D. GROSS, M. D., PROFESSOR OF SURGERY IN THE UNIVERSITY OF LOUISVILLE.

### Case 1.—*Strumous Ophthalmia.*

The patient, a girl, aged ten years, always enjoyed good health until about three years ago, when she began to suffer from inflammation of the eyes, from which she has never been entirely free since. The prominent symptoms, all along, have been intolerance of light, larcrymation, and adhesion of the edges of the lids in the morning. There has been but little discoloration, at any time, so far as we can ascertain, of the eye; and the conjunctiva, this morning, is nearly of the natural aspect, except that there is here and there a straggling vessel, extending inwards towards the cornea, and carrying an undue quantity of blood, thus giving the membrane a slightly congested appearance. Each cornea retains its natural transparency, except at one or two small points, where there is some degree of haziness, dependent upon the presence of plastic matter. The opacity is quite superficial, and apparently of recent formation. The conjunctival lining of the lids is abnormally red, but in a less degree than in ordinary inflammation. It is free from granulations and hypertrophy. The edges of the lids are somewhat discolored and inflamed, and the Meibomian follicles are, perhaps, a little too much developed. No pus or mucus is seen upon the eye, or the lids, but along the eyelashes are a few thin, delicate, bran-like scales, so common in this form of inflammation. The patient, it will be noticed, is unable to throw her head up, so as to face the light; on the contrary, she screens the eyes with her hands, to prevent the light from falling upon the retinæ.

Such are the appearances and condition of the affected organs.

If you will now look at the patient, you will observe that she is in delicate health, and that she has light hair, blue eyes, with long lashes, a fair complexion, tumid lips, and a protuberant abdomen. The extremities are habitually cold, owing to the languid state of the circulation; the pulse is feeble and rather slow for one of her age, and there is a slight enlargement of several of the lymphatic ganglions on the right side of the neck, along the inner margin of the sterno-mastoid muscle. The bowels are regular, and the appetite and sleep good. I cannot ascertain that the mind is particularly sprightly. The child has evidently not had the advantages of education, which has been interrupted by the state of her eyes. Her parents are both living, and they appear to be free from scrofulous disease.

The *symptoms*, in this case, are, it will be perceived, of a mild character. In your practice, you will often meet with the disease in a much more severe and distressing form, attended with the greatest possible intolerance of light, excessive lacrymation, and violent pain. I constantly see cases in which the smallest ray of light is productive of the keenest suffering, and where, consequently, the patient uses every possible precaution to prevent its intrusion. For this purpose he generally, if he be a child, as is commonly the case, creeps into the darkest corner of his chamber, where he covers his eyes with his hands, or buries his head in a pillow, or, perhaps, in the lap of his mother. In this condition he often remains for hours, afraid to change his posture, lest the light should meet his eyes, and thus increase his distress. Children thus affected frequently experience an aggravation of all their suffering even from the light of the moon and of the stars, such is the excessive sensibility of the retina. Photophobia, then, or intolerance of light, is a most important diagnostic symptom in this affection, and one which no practitioner should disregard.

The lacrymation also exists in various degrees, under different circumstances, and in different individuals. In most of the cases that have fallen under my observation, it has been, at one stage or other of the complaint, a prominent symptom. Exposure to light and cold always increases it. The tears are usually hot and scalding, and their discharge is almost always attended with temporary relief.

Sometimes they are so acrid as to irritate the cheeks, causing them to becomered and swollen. The quantity of lacrymal fluid that is thus evacuated in the twenty-four hours may amount to several drachms.

It is rare, in strumous ophthalmia, to witness a copious discharge of mucus, or of muco-purulent fluid. Even when there are excessive photophobia and great lacrymation, it is seldom that there is much secretion of this description; often, indeed, not enough to agglutinate the edges of the lids. In this respect, scrofulous inflammation forms a striking contrast with some of the other varieties of ophthalmia, in which an immense quantity of mucus, or of mucus and pus, is discharged during the height of the morbid action, and even during its declension.

We have seen, in the patient before us, that there is but little redness in the conjunctiva. This is another important symptom in this variety of ophthalmic disease. In ordinary inflammation, discoloration of this membrane is a constant occurrencc, and, generally, it is so conspicuous as to attract, at once, the attention both of the physician and of his friends. In strumous inflammation the vessels observe a straggling arrangement; they are seldom very turgid, and they extend from the circumference of the ball inward towards the cornea, where they are often congregated into little groups, or clusters, beautifully interlacing with each other. When the disease is violent, or of long-standing, the vessels occasionally pass over the cornea, either singly or in parallel lines, separated by narrow intervals. In ordinary ophthalmia the vessels are extremely numerous, and lose, so to speak, their individuality. In a word, there are hundreds where there is one in strumous ophthalmia.

Another important symptom in this form of ophthalmia is the existence of little minute vesicles at the margin of the cornea, occurring either separately or in groups, and varying in size from the smallest perceptible speck to that of an ordinary pin's head. They contain each a minute quantity of serum, and they are frequently encircled by a delicate plexus of vessels, which impart to them a very beautiful and interesting appearance. Their shape is globular, ovoidal, or angular. Sometimes they exist partly on the sclerotica and partly on the cornea. They are witnessed in no other form of ophthalmia, and hence they are of great value as a diagnostic sign.

Strumous ophthalmia seldom continues long without giving rise to opacity of the cornea. This effect, like some of the others that have been mentioned, presents itself in different degress, from the slightest haziness of the part to complete opacity. In the latter case, it is always to be greatly dreaded, inasmuch as it is generally followed by total blindness. Its occurrence should always, if possible, be prevented, not only because it is liable to impair the sight, but because it must necessarily, when irremovable, disfigure the eye and injure the expression of the countenance. It need hardly be added that the immediate cause of this phenomenon is a deposit of lymph into the structures of the cornea.

Ulceration of the cornea is another effect of this variety of ophthalmia, and one, in fact, of frequent occurrence. It often begins at an early stage of the disease, and may proceed, with more or less rapidity, until it extends through the entire thickness of the membrane. The most common form of the ulcer is that of a dimple-shaped depression, with smooth and rather sharp edges, the surface looking as if a piece had been scooped out of it. Generally, the ulcer has a hazy appearance, but not unfrequently it does not differ in its color from that of the adjacent parts, and hence, unless the cornea is examined with great care, while the light is falling upon it at a particular angle, the disease may readily escape detection. Sometimes several such ulcers exist upon the eye, forming either simultaneously, or in pretty rapid succession. If permitted to progress, they occasionally extend through the different layers of the cornea, as far as the anterior chamber of the eye, the humor of which may perhaps escape through the abnormal opening, or, what is more common, the opening is closed up by the membrane of the aqueous humor, or even by the iris.

The pain attendant upon strumous ophthalmia is sometimes intense, while at other times it is very insignificant, if not wholly absent. In confirmed cases, it is always aggravated by the slightest exposure of the affected organ to the light, by medicated applications, by disorder of the bowels, by indulgence in eating, by rough contact, and by various other circumstances unnecessary to be mentioned. Occasionally it is situated deeply in the ball of the eye, in the orbit, or at the base of the anterior lobes of the brain; sometimes it affects merely the lids and brows; occasionally it is most severe in the temple, forehead, or cheek. It may be sharp, shooting, or darting; dull, heavy, or aching; throbbing, or pulsatile; continued, or intermittent. Not unfrequently it assumes a neuralgic character, recurring periodically, like neuralgic pain in other parts of the body. Whatever may be its character, it is often so severe as to deprive the patient of sleep and appetite, and, indeed, of all comfort, for days and weeks together.

Strumous ophthalmia is rarely attended with any tumefaction of the lids. On the contrary, these structures usually retain their normal shape and size; but, in consequence of the excessive intolerance of light, they often present a remarkably drooping appearance, owing to the manner in which they are drawn over the eyes. When the disease is very protracted, the edges of the lids frequently become inverted, so that the ciliæ impinge constantly against the cornea, thereby inducing opacity of this membrane, great increase of pain, and additional inflammation. Although, in general, there is an absence of swelling of the lids, yet you will occasionally find this symptom to exist in a very marked degree. This is especially apt to happen in young children of a leucophlegmatic habit, with a thick upper lip, a tumid belly, and a soft flabby tongue, along with great derangement of the digestive apparatus. The whole system, in such cases, seems to be surcharged with strumous disease, which, in consequence, it is extremely difficult to dislodge from the eyes, which frequently become, so to speak, its victims.

In many cases there is an appearance of little vesicles on the cheeks, the inferior lids, round the nose, or on the lips. Their number varies from two or three to several dozens; their volume rarely exceeds that of the head of a small pin; and their contents are of a serous character. They have a whitish, almost pearly, aspect, are usually discreet, though often closely grouped together, and rest, as it were, upon a reddish base. These vesicles, according to my observation, are most common in children of a deeply-marked strumous habit, and they seldom manifest themselves until after the inflammation has made considerable progress.

I always look upon them with a feeling of misgiving in regard to the ultimate issue of the case; for their presence almost invariably denotes great obstinacy in the morbid action, and proportionate difficulty in effecting a prompt and permanent cure.

From the symptoms which have now been detailed to you, and from the appearance of the patient before you, you can hardly fail in establishing the *diagnosis* of this affection in any case that may fall under your observation. The excessive intolerance of light, the unwonted lacrymation, the absence of redness in the conjunctiva, together with the peculiar straggling arrangement of its vessels, the want of tumefaction, and the manner in which the lids are drawn over the ball of the eye, are signs which, once observed, can never be mistaken. Add to these phenomena the fact that the disease usually arises insiduously and without any assignable cause; the strumous appearance of the features; the coldness of the extremities; the tumid condition of the belly; the formation of vesicles on the face, and various other evidences of the strumous diathesis, and all doubt respecting the true nature of the case must instantly vanish. Indeed, no practitioner, unless he is culpably ignorant of ophthalmic diseases, can possibly commit an error of this kind.

The *prognosis* in this disease must necessarily be influenced by various circumstances, as, for example, the progress and extent of the morbid action, the state of the patient's health, and the nature of our remedies. In the milder forms and earlier stages of the malady, and under proper management, recovery of the affected organ may generally be reasonably predicted. But, under opposite circumstances, the worst consequences may, not unfrequently, be looked for. Ulceration of the cornea often extends, despite our remedies, to a great depth, and sometimes even to complete perforation; an event which is sure to be followed by permanent impairment, if not total loss, of sight. Superficial opacity, even when it is diffused over the greater portion of the cornea, is generally readily amenable to treatment, but when it involves several of the layers of the membrane, or when a considerable period has elapsed since its formation, or, in other words, when time has been permitted for the organization of the lymph, upon the presence of which the opacity depends, then the case will necessarily be unpromising, both as it respects the future appearance of the eye and the amount of vision. It is fortunate that strumous inflammation of the eye rarely terminates in gangrene of any of the structures of this organ. Such an event, judging from my own observation, is extremely infrequent.

Of the exciting *causes* of this disorder very little is known with any degree of certainty. Very frequently its origin is ascribed to circumstances which have no agency whatever in its production. Sometimes it is directly traceable to external injury, as a blow, or a wound; in many cases it is apparently brought on by long exposure of the eye to a strong light, or by excessive fatigue of the organ, induced by reading, writing, or sewing. Suppression of the cutaneous perspiration is probably another, if not a frequent, cause of the disease. In young girls, I have occasionally seen it connected with irregularity of the menses, but whether as a cause or an effect has not always been apparent. Perhaps the most common cause of all is derangement of the digestive apparatus. Whenever the predisposition exists, as it always does in this affection, almost any thing, however trivial, may bring on an attack.

The disease may be limited to one eye, or it may occur in both, either simultaneously or successively. I do not deem it necessary here to insist upon the minute, and, as I conceive, unmeaning divisions and subdivisions of strumous ophthalmia laid down by systematic writers on the diseases of the eyes. Such an arrangement can subserve no useful purpose in practice, and would be entirely out of place in a course of elementary teaching. It is sufficient to say that, in nearly every instance of this complaint, there is an involvement of the conjunctiva and cornea, if not also of the sclerotica and iris, and not simply of the conjunctiva and cornea, or of one of these structures alone, as one might suppose by reading the books, and neglecting observation. In all cases, the retina is either inflamed or morbidly sensitive, as is evinced by the excessive intolerance of light attending the malady.

The age at which this disease occurs is an important circumstance in its history. It is extremely rare for it to begin after the period of puberty, and in no instance have I witnessed its outbreak in middle or advanced life. It is emphatically a malady of infancy and early childhood. According to my observation, it rarely shows itself before the age of eighteen months, or two years. It occurs in both sexes, and in every rank and condition of life, but more frequently among the poor, ill-fed, and ill-clothed, than among the refined and wealthy. The offspring of the consumptive, and of those who have suffered from tubercular disease of the spine, hip, arachnoid membrane, and lymphatic ganglions, are most liable to be affected.

From what I have said to you, on various occasions, concerning the *treatment* of this variety of ophthalmic disease, it is not necessary that I should, this morning, enter fully into the consideration of the subject. You will recollect that the great remedy is quinine, either alone, or in union with other means. I am very certain, from my experience in the management of this disease, that quinine deserves to be placed at the head of all other remedies in this variety of scrofulous affections, and yet, in making this remark, it is necessary to introduce a proviso, lest you should be thereby induced to invest it with a degree of confidence to which, valuable as it is, it is not entitled. What I wish to say is simply this, that this medicine will, if properly administered, that is, with due regard to the patient's system and other circumstances, produce the most prompt and salutary effects; while, if these precautions be neglected, it will either prove useless, or even cause mischief. There are, according to my experience, two distinct classes of strumous eye-cases. In the one, the patient is pale and thin, with a languid circulation, and cold extremities; in the other, he is stout and robust, the cutaneous circulation is active, and the hands and feet are habitually warm. Other points of dissimilarity readily suggest themselves, but these it is unnecessary to point out, as the ditinction which I wish to establish must be sufficiently apparent. Now, to treat such cases alike would be a palpable absurdity. It is only by properly discriminating between them that you can expect to arrive at a satisfactory result, as it respects the employment of this important therapeutic agent. Hence, one practitioner will often mismanage a case which another, having more judgment and more experience, will promptly cure, the disease, perhaps, disappearing as if by magic. Let us pursue this idea a little farther.

In the commencement of my treatment in both forms of the complaint, I usually prepare the system by the exhibition of a moderately brisk cathartic of calomel and rhubarb, to clear out the bowels and correct the secretions. If I have reason to suspect that there is much acid in the alimentary canal, I generally combine with the cathartic a few grains of bicarbonate of soda. Thus, a most effectual beginning is made in the treatment of the disease. If the case comes under the first division, that is, if the patient is pale and thin, and is habitually laboring under cold extremities, I now begin the use of quinine, seldom alone, but commonly in combination with sulphate of iron, tartar emetic, and opium, in quantities proportionate to the age and strength of the individual. For a child of ten years, the age of our patient, a grain and a half of quinine, one grain of iron, the twelfth of a grain of antimony, and the eighth of a grain of opium, carefully mixed, will be a suitable dose, repeated every eight hours, or, if the symptoms are urgent, every six hours, or four times in the day and night. If pills or powders are offensive to the patient, the articles may be given in solution, substituting laudanum or morphia for the opium. Where there is a highly-marked strumous diathesis, I sometimes use the iodide of iron instead of the sulphate, but in most instances I give the latter the preference. Tartar emetic I rarely omit in any case, from the fact that I regard it as one of the most valuable remedies we possess in the treatment of scrofulous disease, both of the eye and of other parts of the body. It is a powerful controller of capillary action, and at the same time a most potent sorbefacient: hence it is particularly applicable in all cases attended with deposits of coagulating lymph. The opium allays pain, renders the eye more tolerant of light, and prevents the antimony from irritating the stomach and bowels. The quinine and iron, whether in the form of sulphate or iodide, are powerful tonics; they improve and invigorate the digestive organs, increase the fibrin and coloring matter of the blood, equalize the circulation, and thus augment the temperature of the extremities, and powerfully aid in correcting the strumous diathesis. By means of these remedies, assisted by a proper diet and due attention to the bowels and secretions, almost any case of scrofulous ophthalmia may, in the class of patients under consideration, be effectually relieved, and that, too, in a comparatively short period.

My favorite cathartic, in these cases, is calomel in combination with rhubarb; to which I occasionally add a few grains of soda, especially if there is reason to suspect the existence of a redundancy of acid in the alimentary canal. In a child from three to five years of age, about two and a half grains of the former, to five or six of the latter, should be given every fourth night. Occasionally the calomel may be advantageously replaced by blue mass; or, in infants, by the grey powder.

When the skin is dry and inactive, the tepid bath may occasionally be employed, or, what is better, the body may be sponged once a day with tepid salt-water, followed by frictions with a coarse dry towel. Flannel should be worn next the surface, both in summer and winter; and the greatest attention should be paid to the preservation of the temperature of the feet. Where they are habitually cold, they should be plunged, twice a day, for a few minutes at a time, into cold water, and then well rubbed with a dry cloth. It is a great mistake, in such cases, to bathe the feet in warm water, with a view to the restoration and maintenance of their temperature.

In the second class of cases, where the general health is apparently but little impaired, where the countenance is florid instead of being pallid, and where the extremities are, for the most part, warm, the quinine is most advantageously conjoined with sulphate of magnesia and tartar emetic, in the form of the saline and antimonial mixture. The following is the formula which I commonly employ under these circumstances:—

| | | |
|---|---|---|
| ℞. | Quininæ sulph. | gr. xxv. |
| | Magnesiæ " | ℥i. |
| | Antim. et potassa tartr. | gr. j½. |
| | Aquæ destillatæ. | ℥ii. |
| | Syr. zingib. | ℥i. |
| | Tinct. opii. | gtt. xxx. |
| | Acid. sulph. ar. | gtt. xxv. |
| | M. | |

Of this mixture, which, considering its ingredients, may be regarded as an exceedingly elegant one, the dose is about one drachm for a child four or five years of age, repeated every four, five or six hours. If it induce vomiting, or nausea beyond a few minutes, it should be diminished. When the inflammation is very severe, I often omit the quinine until the disease has assumed a subacute character, and in that case also I occasionally take blood freely from the arm, or by leeches from the anterior part of the temples, within an inch from the outer commissure of the lids. In the strong and robust, iron, in every form, is totally inadmissible. The diet, too, must be more restricted, and more active purgatives are required. Indeed, the treatment should be strictly antiphlogistic, as much so as in inflammation of the eye from ordinary causes.

As to counter-irritation, collyria, and salves, so much used by practitioners, they cannot be too much or too pointedly condemned. Except in the latter stages of the complaint, in some rare circumstances, it is difficult to conceive of any case in which they would be likely to be beneficial. I am only speaking my real sentiments when I declare that I know of no class of remedies which have done more mischief, or which are so well calculated to fret and annoy the patient, and to support and perpetuate the morbid action. Setons are abominably filthy and painful, and should be discarded from this branch of surgery; tartar emetic ointment and croton oil cause injurious irritation; in short, the only eligible article of this class of remedies is a small blister behind the ear, or, what is preferable, because more easily managed and more permanent, a very small issue, in this situation, made with the Vienna-paste. This, when the eschar is detached, may be dressed, twice a day, with a little adhesive plaster, and will furnish a free discharge for several weeks, when, if necessary, it may be easily re-opened by the application of a little more paste, or some irritating ointment.

The best collyrium, undoubtedly, is a solution of nitrate of silver; but, to answer the purpose, it should be very weak, and not be used until the inflammatory action is greatly diminished, when it may assist in expediting and perfecting the cure by contracting the enlarged vessels of the conjunctiva and cornea, by allaying the morbid sensibility of the eye, and by promoting the absorption of effused lymph. The strength, at first, should rarely exceed half a grain to the ounce of water, which may be gradually increased to a grain, or even twice, thrice, or four times that quantity, according to the circumstances of the case. Sulphate of zinc, acetate of lead, Goulard's extract, and similar articles are worse than useless.

When there are ulcers on the cornea, and they do not yield to the remedies already enumerated, they should be touched, as lightly as possible, once every other day, with the point of a camel-hair pencil wet with a weak solution of nitrate of silver, say three grains to the ounce of water; or, with the nitrate of silver in substance; the former, however, being generally preferable, unless the ulcer is in a phagedenic or gangrenous condition, when the latter should take the place of the solution, as being more prompt and efficacious in its action.

The only eye-salve which I ever employ in this affection is the ointment of the nitrate of mercury, in a very dilute state; generally in the proportion of about ten grains to the drachm of prepared lard. The ointment of the shops is entirely too strong, and can never be used without the risk of materially augmenting the morbid action. Diluted in the manner above stated, it may be advantageously applied in all cases attended with great relaxation of the vessels of the affected part, opacity of the cornea, and adhesion of the lids. The proper way to use it is to annoint the edge of the lower lid with a small pencil, dipped in the salve, every night at bedtime. When the salve is stiff, it should be previously warmed, otherwise it will not be likely to adhere. Thus employed, a very small quantity, a portion not larger than half a grain of rice, will suffice.

Some patients experience great relief from bathing the forehead, face, and temples, frequently with warm water, pretty strongly impregnated with common salt; while others derive most benefit from bathing with cool, cold, or hot water. In all cases the best plan is to permit the patient to consult his own feelings in the use of this remedy.

It need hardly be added that the eyes should always be carefully protected with a green shade; but on no account should the patient be allowed to wear green glasses, or, what is still more abominable and injurious, goggles. Such a practice, indeed, cannot be too much deprecated. The same remark is applicable to compresses and bandages. I have seen numerous cases in which irreparable mischief has been done by the protracted use of these articles. The true, legitimate practice consists in protecting the affected organs in such a manner that, while they are sufficiently screened from the light to render the patient comfortable, they shall have the full benefit of cool air. As the morbid action declines, more and more light should gradually be admitted, until at length they receive their accustomed supply. It should never be forgotten that light is the natural stimulus of the eye, and that by withholding this stimulus for too long a time, the organ may become morbidly sensitive; just as the stomach becomes irritable and unable to perform its functions when it is for a long time deprived of food.

Finally, I may state to you that I have rarely derived any essential benefit, in the treatment of any form of scrofulous ophthalmia, from iodide of potassium, so much vaunted by some practitioners. Formerly I was in the habit of prescribing this article quite frequently, but it so often totally disappointed my expectations that I have, of late years, laid it entirely aside. In obstinate cases, you will occasionally obtain benefit, especially in weakly children, requiring an alterant and tonic, from the exhibition of bichloride of mercury, in very minute doses, as the twentieth or twenty-fifth of a grain, in union with Huxham's tincture of barks. I am well aware that the salt in this prescription undergoes some chemical change; but this renders it, perhaps, only the more efficacious. It is not necessary to carry the remedy to the extent of ptyalism to obtain its full effects. Indeed, such an occurrence should always be carefully avoided.

Where there is hemi-crania, or excessive circum-orbitary pain, anodynes are necessary, particularly at night, both to allay suffering and to procure sleep. Under such circumstances, some practitioners are in the habit of applying belladonna ointment to the affected parts, and in some cases I have found the remedy of service, but, in general, it has disappointed me.

During the latter stages of the disease, the patient should take, daily, gentle exercise in the open air, as a means powerfully calculated to improve his general health, and to invigorate his constitution. In all cases, the greatest care should be taken to avoid exposure and indulgence of the appetite and passions. As another powerful means of guarding against relapse, a moderate use of the remedies, above mentioned, should be persisted in for a considerable time after all disease has apparently vanished.

---

CASE 2.—*Hydatid Tumor of the Arm; Excision; Cure.*

WILLIAM GAR, a stout, healthy-looking Irishman, aged twenty-five years, applied at my office, a few days ago, on account of a small tumor on the right arm. When first noticed, about six months ago, it was not larger than a hazel-nut, rather hard, and free from pain. Its discovery seems to have been altogether accidental. The man is a common laborer; and, although rather intemperate, has always enjoyed good health. Supposing that the case would be of interest to you, I have requested him to come here to-day, and I now show you the affected part. It will be perceived that the tumor, which is of a rounded, elongated shape, occupies the outer part of the arm, and that it is apparently lodged in the substance of the deltoid muscle. It is about the volume of a large almond with its shell, somewhat movable, especially at its inferior extremity, and of a firm, fibrous consistence, with a slight degree of elasticity. In fact, the sensation which it imparts to the finger is very much like that of an indurated lymphatic ganglion; a circumstance which, were the site not unusual, would suggest the idea that it might be a body of that kind. Within the last two weeks, the tumor has been somewhat painful, especially after exercise, and the pressure made in examining it is productive of slight uneasiness. The skin is perfectly healthy, and there is no disease in the neighboring parts. It is proper to remark that the tumor is increasing in size, though not rapidly, and that the patient is not aware of any thing that could have produced it.

In regard to the diagnosis of this tumor, it is impossible to offer even a conjecture. If it were situated in a region of the body where lymphatic ganglions occur, I should unhesitatingly pronounce it to be nothing but a structure of this kind, in a state of induration and enlargement; but, inasmuch as this is not the case, such a conclusion would be unwarrantable. That it is not a fatty tumor is apparent from its consistence and rapid progress. Growths of the latter description are always soft, especially when small, and of slow development. I can not suppose that it is a sebaceous tumor, because it is hard, and too irregular in its outline to justify such a belief, to say nothing of the fact that the sebaceous, like the adipous tumor, is always remarkably tardy in its formation. It may be an encrysted tumor; but here, again, we are met by the fact that there is an absence of some of the characteristics of this class of morbid growths, particularly elasticy and fluctuation. Nor is it likely to be malignant, and yet upon this subject it behooves me to be somewhat guarded. We may assume that it is benign from the circumstance, chiefly, that the man is not more than twenty-five years of age, and that there is an absence of the sharp, lancinating pain so conspicuous in malignant disease. Possibly, the growth may be fibrous; for it certainly has a good deal of the feel of such a tumor; but here, too, our diagnosis is at fault.

The above remarks, which, were the occasion suitable, might be greatly extended, will, I trust, not be lost upon you; inasmuch as they will serve to show you how imperfect are our means of diagnosis in certain forms and stages of morbid growths. If the tumor were not so firm, I might use the exploring needle; for, in this way, I might, perhaps, be able to ascertain the nature of its contents; but its solidity forbids such a mode of examination; and I shall, therefore, proceed to remove it; hoping, after a careful inspection, to be able to determine its structure, and to refer it to its appropriate head.

In performing the operation, I shall make a simple vertical in-incision, when, reflecting the edges to their respective sides, I shall, probably, be able to enucleate the tumor by a rapid dissection, or merely by using the handle of the scalpel.

The above remarks having been made, the operation was proceeded with- In carrying the incision over the tumor, the knife penetrated it near its middle, and immediately there gushed out a small quantity of thin purulent fluid, inducing the belief that the swelling, after all, was merely a diseased lymphatic ganglion. Continuing the dissection, however, I soon found that there was a distinct cyst, which, being collapsed, required some pains to separate it from the fibres of the deltoid muscle, in which it was embedded. After the removal was completed, Dr. Richardson, who had kindly assisted in the operation, picked up a small body from the floor, which, upon examination, proved to be a globular hydatid, not more than about six lines in diameter, and furnished with a distinct sucker. The cyst was very soft and thin, smooth internally, and filamentous externally. Hardly any blood was lost in the operation. The parts were approximated by adhesive strips; and, at the end of a week, the greater portion of the wound had united by the first intention.

Considering the great rarity of this form of tumors, it is not surprising that we should have been unable to determine its diagnosis before its removal. Only a few examples of a similar kind are upon record. In some of the internal organs, as the liver and ovary, hydatids are occasionally observed, both in the human subject and in the inferior animals; but they are very uncommon in the other viscera, and also in the cellular tissue, whether beneath the skin or among the muscles. Of their cause and mode of formation nothing is known. After having attained a certain age, they sometimes lose their ostality, and thus becoming a source of irritation, induce suppuration in the surrounding parts, followed occasionally by their elimination. The probability is that this process had already commenced in the case of Gar, as was evidenced by the escape of pus on puncturing the cyst in the dissection, although the hydatid itself was apparently unchanged. The great consolation, connected with the tumor, is, that it is benign in its character, and, therefore, unable to return.

